# Disparate Dynamics of Gene Body and *cis*-Regulatory Element Evolution Illustrated for the Senescence-Associated Cysteine Protease Gene *SAG12* of Plants

**DOI:** 10.3390/plants10071380

**Published:** 2021-07-06

**Authors:** Emil Vatov, Uwe Ludewig, Ulrike Zentgraf

**Affiliations:** 1 Center for Plant Molecular Biology (ZMBP), University of Tübingen, Auf der Morgenstelle 32, 72076 Tübingen, Germany; E.Vatov@uni-hohenheim.de; 2 Institute of Crop Science, Nutritional Crop Physiology, University of Hohenheim, Fruwirthstr. 20, 70599 Stuttgart, Germany; u.ludewig@uni-hohenheim.de

**Keywords:** *SAG12*, senescence, promoter, gene body, evolution, phylogeny, *cis*- and *trans*-regulators of transcription

## Abstract

Gene regulation networks precisely orchestrate the expression of genes that are closely associated with defined physiological and developmental processes such as leaf senescence in plants. The *Arabidopsis thaliana* senescence-associated gene 12 *(AtSAG12)* encodes a cysteine protease that is (i) involved in the degradation of chloroplast proteins and (ii) almost exclusively expressed during senescence. Transcription factors, such as WRKY53 and WRKY45, bind to W-boxes in the promoter region of *AtSAG12* and play key roles in its activation. Other transcription factors, such as bZIPs, might have accessory functions in their gene regulation, as several A-boxes have been identified and appear to be highly overrepresented in the promoter region compared to the whole genome distribution but are not localized within the regulatory regions driving senescence-associated expression. To address whether these two regulatory elements exhibiting these different properties are conserved in other closely related species, we constructed phylogenetic trees of the coding sequences of orthologs of *AtSAG12* and screened their respective 2000 bp promoter regions for the presence of conserved *cis*-regulatory elements, such as bZIP and WRKY binding sites. Interestingly, the functional relevant upstream located W-boxes were absent in plant species as closely related as *Arabidopsis lyrata*, whereas an A-box cluster appeared to be conserved in the Arabidopsis species but disappeared in *Brassica napus.* Several orthologs were present in other species, possibly because of local or whole genome duplication events, but with distinct *cis*-regulatory sites in different locations. However, at least one gene copy in each family analyzed carried one W-box and one A-box in its promoter. These gene differences in *SAG12* orthologs are discussed in the framework of *cis*- and *trans*-regulatory factors, of promoter and gene evolution, of genetic variation, and of the enhancement of the adaptability of plants to changing environmental conditions.

## 1. Introduction

Plant evolution depends on variation in the genetic pool of a population and natural selection acting to establish beneficial traits or to prune harmful mutations. Plants encounter a large variety of stress conditions throughout their life cycle, ranging from herbivory and disease to soil imbalances such as acidity, salinity, drought, nutrient deficiencies, and fluctuating weather conditions. These affect plant growth and specific organs or tissues and determine the efficiency with which the plant can utilize its genetics to survive and reproduce. Plants that withstand more adverse conditions have a reproductive advantage under such conditions, leading to the selection of various defense and avoidance traits. In this sense, long lasting unfavorable stresses, e.g., drought, salinity, and nutrient deficiency, lead to the premature activation of senescence as an exit strategy to guarantee the production of offspring even under such harsh conditions. However, this is often combined with the tradeoff in seed number and quality [1]. Under stress-free conditions, leaf senescence is governed by the age of the leaves and the whole plant [2]. It aims to maximize the usage of the plant’s resources for growth and the sake of the next generation by an efficient recycling of carbon, nitrogen, and mineral resources out of the senescing tissue into developing parts of the plant such as new leaves or fruits and seeds. Very complex gene regulatory networks are in place to integrate all kinds of stress responses into this developmental process [3]. Although such networks are conserved, even among distantly related species, individual regulatory genetic factors are often distinct [4,5]. 

In general, gene expression is driven by *cis*-elements on the DNA or *trans* factors binding to them. Signor and Nuzhdin [5] have pointed out that variation in *trans* factors can potentially arise anywhere in the genome and often provide larger intraspecific contributions to gene expression variance, whereas *cis*-regulatory differences lead more prominently to divergence between species. Under stabilizing selection, *cis-trans* compensation probably serves to re-stabilize gene expression levels, despite the presence of regulatory variation [5]. When one tries to distinguish *cis* and *trans* effects on gene expression, *cis*-regulatory changes appear less pleiotropic and are more often found in genes that do not have a direct influence on other genes. These can frequently be detected in peripheral non-hub positions of a gene regulatory network in genes that have structural, rather than regulatory, functions [5]. In concordance, a study in maize and sorghum [6,7] has indicated that differentially expressed genes, which are part of the central “subnetwork” involved in sensing cold exposure, are relatively well conserved between species, whereas the peripheral “subnetwork” exhibits higher variation. 

*SAG12* is a senescence-associated gene coding for a cysteine protease that is involved in the degradation of chloroplast proteins in *Arabidopsis thaliana* leaves. *SAG12* is almost exclusively activated during senescence; therefore, the quantitative expression of *SAG12* is often measured, amongst others, as a marker to quantify the progression of leaf senescence [8,9,10]. When fused to GFP, this cysteine protease has been discovered to be localized to so-called senescence-associated vacuoles (SAVs), which become visible in senescent leaves and are involved in the degradation of chloroplast proteins [11,12]. Since 1995, the *SAG12* promoter sequence has been used successfully to delay senescence in tobacco plants, when fused to the *isopentenyl transferase* gene, which encodes a key enzyme of cytokinin biosynthesis [13]. In this case, Gan and Amasino used approx. 2.2 kbp upstream of the start codon as functional promoter whereas Otegui et al. [11] have used a 2.8 kbp region. 

In 1999, Noh and Amasino [14] described two separate regulatory *cis*-acting regions of the *AtSAG12* promoter in detail. They identified a highly conserved regulatory element that was located at position −747 to −570 bp relative to the potential transcription initiation site (TIS) and that was sufficient to promote age-associated gene expression and responsiveness to cytokinin, auxin, and sugar treatments. Furthermore, an enhancer region located at position −1345 to −1181 bp relative to the potential TIS substantially increased the expression of a marker gene, when combined with the −784 to −471 region (relative to TIS). The combination of these two regions was able to induce expression levels close to those of the complete native promoter. However, at that time, Noh and Amasino [14] could not suggest any *cis*-elements responsible for the specific regulation of *SAG12* even though they could detect the binding of an unknown protein to a smaller 32 bp fragment within the −747 to −570 region, which is highly conserved between *A. thaliana* and *Brasscia napus.* A direct interaction of the *AtSAG12* promoter with the senescence-regulating transcription factor WRKY53 was discovered in a DNA pulldown assay by using recombinant 6xHis-tagged WRKY53. The binding to W-boxes present between approx. −1600 bp and −1100 bp relative to the start codon was confirmed by EMSA [15]. Further clarification was achieved by Chen et al. [16], who described a *cis*-regulatory region that contained three complete W-boxes (T/CTGACC/T) and that overlapped the region used by Miao et al. [15]. The three W-boxes appeared to be binding sites for the *trans*-acting transcription factor WRKY45. Based on this experiment, Chen et al. [16] described WRKY45 as a positive regulator of leaf senescence and of *AtSAG12* expression in particular, thus explaining the role of the enhancer region described earlier by Noh and Amasino [14]. Whether and how WRKY53 and WRKY45 act together in the regulation of the *AtSAG12* expression and the role of other possible transcription factors requires further investigations.

Almost all transcription factor families in plants are involved in senescence regulatory processes; however, the families of WRKY and NAC factors, which largely expanded in the plant kingdom, are overrepresented in the senescence transcriptome of Arabidopsis [17]. NAC proteins form one of the largest plant-specific TF super family, and its members are implicated in the regulation of transcriptional reprogramming associated with many developmental processes including senescence [18]. In general, the age- and stress-regulated expression of senescence-associated genes is mediated by a complex gene regulatory network [3]. Among other families of transcription factors, WRKYs and bZIPs are also essential components of this network in which WRKYs target the conserved W-box domain, and bZIPs target sequences with an ACGT core, e.g., A-boxes, C-boxes, G-boxes, and T-boxes [3,19]. However, the exact number of factors and elements regulating *AtSAG12* is unknown, both in *cis* and in *trans*. On average, any Arabidopsis gene is estimated to be targeted by 25–75 binding events with transcription factors [7,20]. *SAG12* genes are also found in other plant species including major crop plants such as oilseed rape, maize, barley, or rice [21], but less is known about their regulation. In *Arabidopsis thaliana* but also in other species, *SAG12* belongs to the large family of cysteine protease genes [21]. Clearly, the coding sequences of functional genes, such as *SAG12*, are under strong evolutionary pressure, as mutations might disrupt the normal function of the protein product. By contrast, gene promoter regions can tolerate a relatively high number of mutations and may slightly shift functional boxes without losing their functionality [22]. As a “peripheral” network gene producing a catalytic enzyme involved in protein degradation, with no known direct influence on other genes, *SAG12* is a good candidate for the accumulation of noticeable variation in *cis* throughout evolutionary timescales. 

In this paper, we discuss the evolution of the promoter region of *SAG12* genes involved in plant leaf senescence. We hypothesize that at least the main regulatory elements of a specific promoter are conserved in related species (although not exactly at the same position) to warrant proper gene regulation. We have compared the coding sequences and the 2000 bp regions upstream of the start codon of genes orthologous to *AtSAG12* in various plant species ranging from aquatic unicellular organisms to monocot and dicot land plants including annuals and perennials. We have found that the cysteine protease gene *SAG12* is highly conserved across the whole plant kingdom but have identified disparate differences in promoter *cis*-regulatory features, suggesting that defined senescence-associated developmental roles are likely to be accomplished by stable gene regulatory networks with distinct components.

## 2. Results

### 2.1. The Arabidopsis thaliana SAG12 Promoter

**Figure 1:**
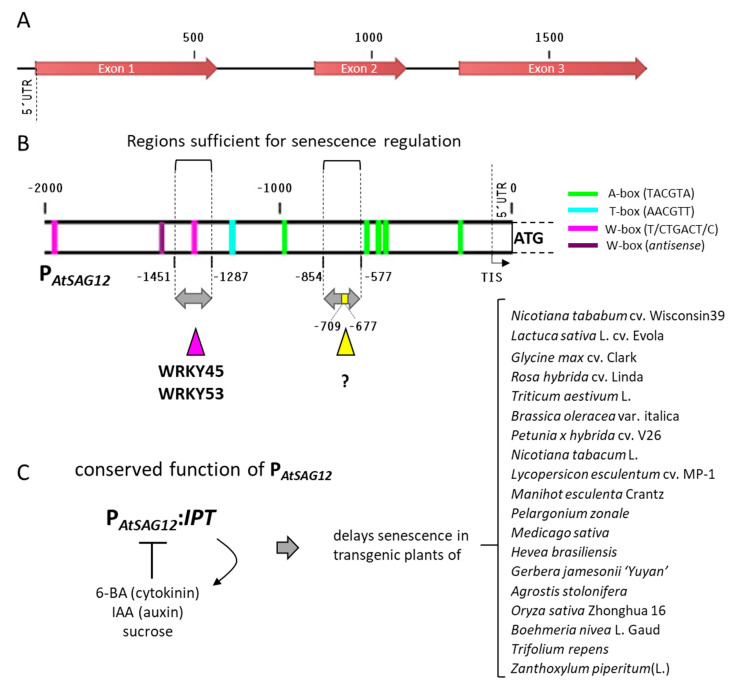
Gene and promoter structure of *AtSAG12*. (**A**) Exon (red arrows) and intron (black line) structure of *AtSAG12* (AT5G45890.1) and (**B**) 2000 bp region adjacent to the ATG start codon, including the 5′UTR, annotated for the presence of W-boxes (T/C TGAC T/C), A-boxes (TACGTA), T-boxes (AACGTT), C-boxes (GACGTA), and G-boxes (CACGTA). W-boxes in sense (Pink), W-boxes in anti-sense (Purple), A-boxes (Green), T-boxes (Cyan). The two regions characterized by Noh and Amasino [14] are indicated by the grey arrows. WRKY45 and WRKY53 were shown to bind to the W-box in the region −1451 to –1287 relative to the start codon (pink triangle) [15,16], which is equivalent to the enhancer region (−1345 to −1181 relative to the TIS) [14], whereas in the 32 bp region indicated in yellow (−709 to −677 relative to the start codon) binding of unknown protein(s) was observed [14]. (**C**) The promoter region of *AtSAG12* was used in a transgenic approach to drive the isopentenyl transferase gene (*IPT*), which leads to an increase production of cytokinins, which then inhibits the *SAG12* promoter activity. This autoregulatory feedback loop delays senescence in many different species indicating a conservation of all functional components. TIS (transcription initiation site), ATG (start codon), 5′UTR (5′untranslated region).

Initially, we mapped the locations of all W-boxes (T/CTGACT/C), A-boxes (TACGTA), T-boxes (AACGTT), C-boxes (GACGTA), and G-boxes (CACGTA) in the Arabidopsis genome and found the *AtSAG12* gene to be suitable for further analysis, because of its well-characterized promoter region and the prominent presence of these *cis*-regulatory elements in it. The protein coding sequence of the *AtSAG12* gene is 1041 bp long and comprises three exons separated by two introns (Figure 1A). The *AtSAG12* promoter (2000 bp upstream of the ATG) contains not only W-boxes that are targeted by WRKY transcription factors [15,16,23], but also T-boxes and A-boxes (Figure 1B) that are potentially targeted in *trans* by bZIP [19] and NAC [24] factors. Interestingly, five A-boxes (TACGTA) are located in the region of −1000 to 0 bp relative to the start codon of *AtSAG12* (Figure 1B). Three of these five A-boxes are located close to but outside of the −748 to −471 bp region relative to the TIS that has been described by Noh and Amasino [14] as the main conserved regulatory region of the *AtSAG12* promoter. This region −748 to −471 bp relative to the TIS identified by Noh and Amasino [14] is equivalent to the region −854 to −577 according to our numbering relative to the start codon. Even though the A-boxes are not part of the regions responsible for senescence-associated expression and are, therefore, most likely not directly involved in this regulation, these elements appear to be overrepresented in the promoter region, and their appearance seems to be conserved as analyzed and described in the following. Moreover, bZIP transcription factors are often involved in plant responses to abiotic and biotic stresses and in age-induced leaf senescence [25], indicating that some, if not all, of these A-boxes might be responsible for the crosstalk of stress- and age-regulated gene expression of the *AtSAG12* gene. Because of the large differences in promoter sequences, these *cis*-regulatory sites were used in the following as markers to trace promoter evolution in a comparison of the promoters of orthologous genes.

Furthermore, we used Softberry Nsite-PL [26,27] to recognize alternative transcription factor binding motifs. The algorithm mapped 28 additional transcription factor binding sites (Supplementary Table S1). Surprisingly, the W- and A-boxes included in our analysis were not recognized. Only two alternative binding sites were detected within the −747 to −570 region relative to TIS used by Noh and Amasino [14]: a PY-box, initially found in *Oryza sativa* and recognized by the OsDOF3 transcription factor, and a 10 bp Z-BAC, initially found in *P. vulgaris*. None of these *cis* elements appears to be responsible for the binding of the uncharacterized protein, as all three are located upstream of the region used for the DNA–protein binding assays (Figure 1B). The enhancer region located −1345 to −1181 bp upstream of the TIS (−1451 to −1287 upstream of the ATG) contained alternative binding sites: a S2 binding site, found in *A. thaliana* and recognized by an unknown nuclear factor; a CCA1 motif, found in *A. thaliana* and recognized by CCA1 transcription factor; a NIT BS II motif, found in *C. vulgaris* and recognized by NIT2; a TAGTCAAC motif found in *P. crispum* and recognized by an unknown nuclear factor. However, whether all these binding sites are really of importance and are bound by the respective factors still has to be analyzed in more detail. So far, no circadian expression has been described for *SAG12*. Noteworthy, the *AtSAG12* promoter region was used in a transgenic approach to drive the *isopentenyl transferase* gene (*IPT*). IPT is an enzyme involved in the production of cytokinin, which then signals back to the *AtSAG12* promoter to inhibit further transcriptional activity. This autoregulatory feedback loop has first been established in transgenic tobacco where it significantly delayed senescence and increased seed production [13]. As yet, the P*_AtSAG12_*:*IPT* construct has been introduced into a wide range of different plants all showing delayed senescence (Figure 1C, Supplemental Table S3). Moreover, senescence-associated expression of *SAG12* orthologs was observed in many different species including rapeseed, poplar, barley, rice, maize, and *Brachypodium distachyon* (Supplemental Table S4), indicating that overall regulation appears to be conserved over the plant kingdom. Therefore, we analyzed the presence and distribution of *cis*-elements in the promoters of *SAG12* genes.

### 2.2. Random and Non-Random Distribution of A- and W-Boxes in the AtSAG12 Promoter Region

**Figure 2:**
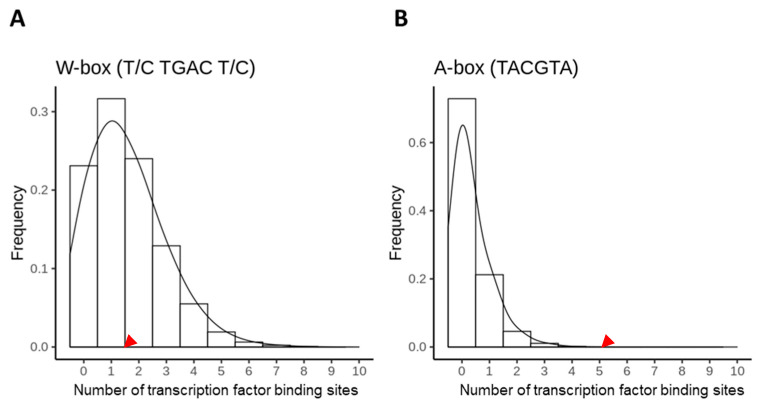
Distribution of (**A**) W-boxes and (**B**) A-boxes in all *A. thaliana* 2000 bp promoter regions. Red arrows indicate the number of corresponding elements observed in the *AtSAG12* −2000 bp promoter region.

To evaluate the possibility that the binding motifs appear at random, we calculated the stochastic occurrence of the WRKY and bZIP transcription factor binding sites based on the distribution frequency of the four nucleotides and dinucleotides in the *A. thaliana* genome, as well as 1507 fragments of 2000 bp with a GC content of 26.4% to 30.4%. These sequences were chosen, as they deviate up to 2% from the GC content of the *AtSAG12* promoter of 28.4%. The GC content of the genome is 36%, making the chance of having a random W-box (T/CTGACT/C) approx. 0.105%. As a result, an average of 2.09 random W-boxes are expected in 2000 bp fragments. This chance drops down to 0.074% within fragments with GC content close to that of the *AtSAG12* promoter, making the expected average to be 1.48 W-boxes per fragment. In the whole genome, we identified 1.60 boxes per 2000 bp fragment, slightly more than the predicted random occurrence. As W-boxes are non-palindromic sequences, we calculated the appearance in both forward and reverse orientations (Watson and Crick strands) and found it to be equal. The occurrence in all 2000 bp upstream regions 5′ to the ATG start codon in *A. thaliana* was on average 1.56 boxes per promoter. The *AtSAG12* promoter had two forward W-boxes and one reverse W-box, values close to the calculated probability for random occurrence (Figure 2A). The calculated stochastic occurrence of an A-box (TACGTA) was 0.011%, i.e., 0.226 A-boxes within 2000 bp fragments. This value increased to 0.017% for fragments with similar GC content as the *AtSAG12* promoter, which suggests 0.344 A-boxes per 2000 bp fragment. Genome-wide, 0.303 A-boxes per 2000 bp genomic fragments were identified, indicating a slight positive selection for A-boxes. Similarly, in promoter regions, 0.349 A-boxes per 2000 bp promoter fragments were identified, but the *AtSAG12* promoter region contains five A-boxes (a 14-fold higher value than those in other promoters), indicating potential positive selection for A-boxes in this promoter region (Figure 2B).

### 2.3. Evolution of the SAG12 Promoter across the Plant Kingdom

**Figure 3:**
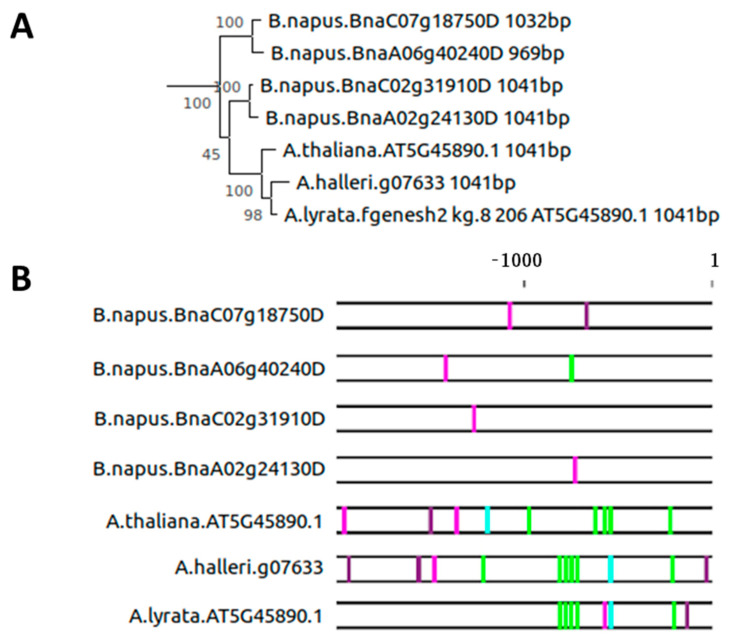
Phylogenic analyses of *SAG12* and its promoter region in Brassicaceae. (**A**) Phylogeny of *SAG12* coding sequence, gene identification numbers, and sequence length. (**B**) 2000 bp region 5′ adjacent to the ATG start codon annotated for the presence of W-boxes (T/CTGACT/C), A-boxes (TACGTA), T-boxes (AACGTT), C-boxes (GACGTA), and G-boxes (CACGTA). W-boxes in sense (Pink), W-boxes in anti-sense (Purple), A-boxes (Green), and T-boxes (Cyan).

We searched the Ensembl Plants database release 45 for orthologs of *AtSAG12* (*AT5G45890*) and selected sequences from 22 species spanning lower to higher plants, perennials and annuals, and temperate to tropical environments (Supplementary Figure S1). We separated the protein coding sequence and the 2000 bp region adjacent to the ATG start codon for alignments and the construction of phylogenetic trees. *AtSAG12* is positioned on a branch that separates all eudicots from the rest of the group (Supplementary Figure S1). Its closest homologs are those of *A. halleri*, *A. lyrata,* and *Brassica napus* (Figure 3A). Interestingly, already, the three Arabidopsis species show remarkable differences in their promoter regions (Figure 3B). A characteristic A-box appears near the start codon of the *SAG12* gene in *A. lyrata* and *A. halleri*, and a nearby reverse-oriented W-box appears that is absent in *A. thaliana*. The hypothetical promoter element of *A. thaliana* containing multiple A-boxes around the −500 bp region seems to be shifted approx. 200 bp upstream and is associated with a T-box or a W-box/T-box complex in *A. halleri* and *A. lyrata,* respectively. The W-box regulatory domain present upstream of position −1000 is completely absent in *A. lyrata*, whereas small positional changes are observed in *A. halleri*. Not only did point mutations occur in potential regulatory elements, but also small insertions and deletions occurred in the promoter region. Softberry NsiteM-PL [26,27] was probed for conserved regulatory motifs within the three Arabidopsis species. Only one binding site was conserved within all three of them, namely the AS1-AS2 BS II (BP Y), with 14 binding sites conserved in two out of the three sequences probed (Supplementary Table S1). *A. thaliana* showed overall higher similarity to *A. helleri* (Supplemental Figure S3). Expression data from the Expression Atlas (http://ebi.ac.uk/ (accessed on 1 November 2020)) of *SAG12* in *A. lyrata* [28] and *A. halleri* confirmed the association with senescence but revealed much lower expression in the flower compared with *A. thaliana* [29]. Furthermore, expression in root tissue was detected in *A. lyrata* but was not detected in several datasets for *A. thaliana* except for one report during the reproductive period under low nitrogen supply [30]. A detailed Expression Atlas was not available for *A. halleri*.

*Brassica napus* is a close relative of *A. thaliana* in the Brassicaceae family and is derived from the interspecific crosses between *Brassica rapa* and *Brassica oleracea* [31]. It contains four orthologs of *SAG12* from genome duplications, two of which lie in the A genome and two in the C genome. These gene sequences align in two A-C pairs, suggesting that each of the two ancestral genomes contain two copies of *SAG12* orthologs on two separate chromosomes (Figure 3A). Interestingly, *B. napus* has almost completely different structures of the 2000 bp promoter region compared with Arabidopsis (Figure 3B). A single W-box is always present in different positions of the four genes, with a single A-box in the promoter of BnaA06g40240D. Given the high similarity of these four coding sequences among themselves and the variation compared with the members in three Arabidopsis species, dynamic diversification must have occurred in promoters during evolution. Wan et al. [32] have detected the expression of the four orthologs during seed development, a developmental stage distinct from senescence. They share more similar expression patterns within a pair than between pairs. Beside the presence of a W-box, the other regulatory elements found in Arabidopsis could not be identified. Softberry NsiteM-PL recognized only two binding sites conserved between *A. thaliana* and one of the two gene pairs (Supplementary Table S1). One of these binding sites was the AS1-AS2 BS II (BP Y). Overall, the two pairs were more similar within a pair than between pairs. In total, eight transcription factor binding sites were conserved in a minimum of three out of the five sequences analyzed.

**Figure 4:**
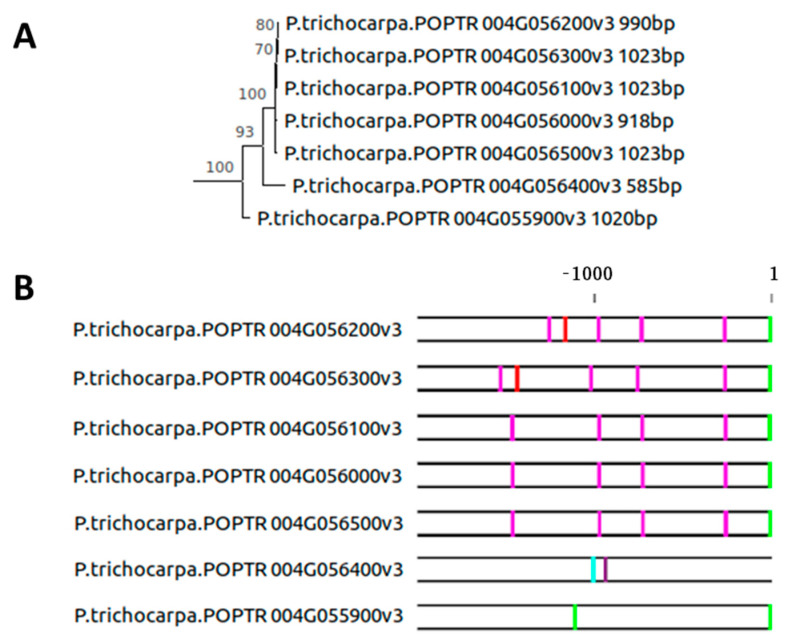
Phylogenic analyses of *SAG12* and its promoter region in *Populus trichocarpa*. (**A**) Phylogeny of *SAG12* coding sequence indicating species, gene identification, and sequence length and (**B**) 2000 bp region adjacent to the ATG start codon annotated for the presence of W-boxes (T/C TGAC T/C), A-boxes (TACGTA), T-boxes (AACGTT), C-boxes (GACGTA), and G-boxes (CACGTA). W-boxes in sense (Pink), W-boxes in anti-sense (Purple), A-boxes (Green), T-boxes (Cyan), and G-boxes (Red).

In the genome of the poplar (*Populus trichocarpa)* tree, seven orthologs of *SAG12* are found in relatively close proximity on chromosome 4 (Figure 4A). Genes POPTR 004G056000v3, POPTR 004G056100v3, POPTR 004G056200v3, POPTR 004G056300v3, and POPTR 004G056500v3 have almost identical coding regions, even though their lengths vary between 918 bp and 1023 bp. The main cause for their differences in length are deletions of various lengths in the 5′ end regions, in POPTR_004G056000v3, and POPTR_004G056200v3. The annotated regulatory elements in their promoters are similar, except for two pronounced differences in POPTR 004G056200v3 and POPTR 004G056300v3 around the −1000 bp to −1500 bp region (Figure 4B). A gene duplication event apparently expanded across the 2000 bp promoters. The POPTR 004G056400v3 copy consists of only 585 bp and differs from the first group of five sequences, residing on a branch of its own in the phylogenetic tree. The reduction in length is attributable to a missing 3′ half of the gene coding sequence, probably leading to a loss of function. As its promoter region is also markedly different, a duplication event either copied only the 5′ half of the gene into a new regulatory context, or the region in which this gene copy resided has undergone some major changes since the duplication event. The lowest branch of the tree contains POPTR 004G055900v3, which also shows marked differences in the organization within its promoter region. Only an A-box in close proximity to the ATG start codon remains, similar to the promoter regions of the other copies. Thus, a single duplication event most likely occurred early in the *Populus* lineage, and after a period of divergent evolution, the region containing one of the two copies (along with its promoter) potentially underwent multiple local duplication events on chromosome 4. The high similarity between the coding sequences and their promoters, given the intense nature of promoter evolution demonstrated above, indicates that these duplication events happened in the relatively recent past. Furthermore, five out of seven sequences share conserved two exon–one intron structure, with differences in length mainly due to changes in intron, 5′UTR, and 3′UTR region lengths. POPTR_004G056200v3 has an additional short intron separating its 3′ exon in two, while POPTR 004G056400v3 has only one intron. Softberry NsiteM-PL found 24 binding sites conserved in at least four out of the eight sequences analyzed (Supplementary Table S1). Only two of these were found in *A. thaliana*: the PY-box from *O. sativa* and the HB05/HB12 from *A. thaliana*. Whereas the expression of POPTR 004G055900v3 has been reported [33], the expression pattern of the other genes, especially during senescence, remains elusive but is expected to be diverse, as deduced from the markedly different promoter regions. In *Populus tremula*, at least one *SAG12* orthologue is expressed during autumn senescence (Supplemental Table S4).

**Figure 5:**
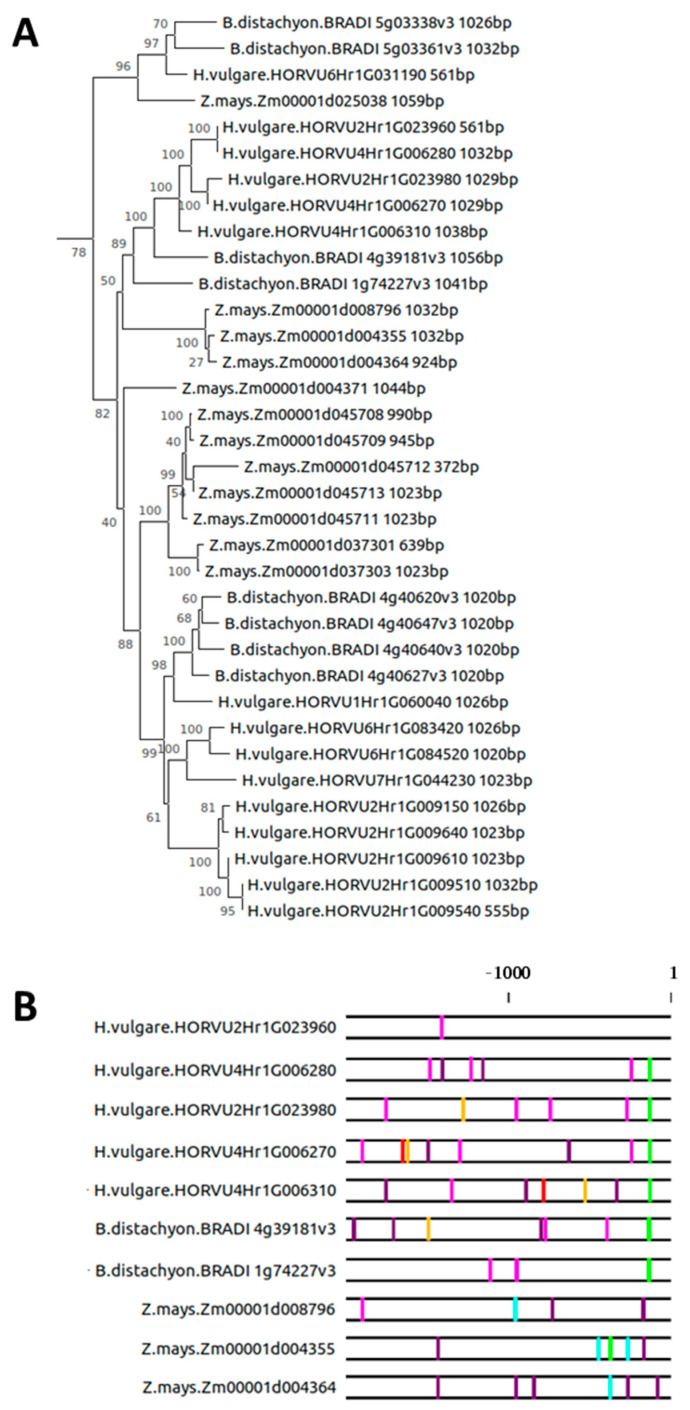
Phylogenic analyses of *SAG12* and its promoter region in *Brachypodium distachyon*, *Hordeum vulgare*, and *Zea mays*. (**A**) Phylogeny of *SAG12* coding sequence indicating species, gene identification, and sequence length and (**B**) 2000 bp region adjacent to the ATG start codon annotated for the presence of W-boxes (T/C TGAC T/C), A-boxes (TACGTA), T-boxes (AACGTT), C-boxes (GACGTA), and G-boxes (CACGTA). W-boxes in sense (Pink), W-boxes in anti-sense (Purple), A-boxes (Green), T-boxes (Cyan), C-boxes (Yellow), and G-boxes (Red). Due to the large number of sequences, we have only shown one of the three main branches of the phylogenetic tree that has at least two copies of the gene in each family.

Another segregation of *SAG12* orthologs on the phylogenetic tree separates the grass members from the rest of the higher plant species (Supplementary Figure S1). From the family of grasses, we included *Brachypodium distachyon, Hordeum vulgare*, and *Zea mays* (Figure 5A), all of which show senescence-associated expression of *SAG12* (see Supplemental Table S4). Two consecutive branching events separate these genes in three main groups that contain representatives from each of the three grass species. This indicates the existence of at least three separate copies of this gene in their most common ancestor. Following gene duplication events, these three ancestral sequences gave rise to several putative genes (Figure 5A). Most of these genes contain markedly different putative regulatory elements (Figure 5B). A characteristic W-box/A-box combination close to the transcription start site was identified in a number of *Hordeum vulgare* sequences and in one of the *Brachipodium distachyon* sequences. This might represent an evolutionary conserved gene regulatory element that existed before the splitting of the two lineages. A combination of 2–6 W-boxes present in a number of promoters from all three species further indicated that at least one copy of the gene might be regulated via WRKY transcription factors, as in *A. thaliana*. No conserved binding motifs were recognized by Softberry NsiteM-PL between *A. thaliana* and the *B. distachyon* and *H. vulgare* promoter regions, with only the GS1b AC box 1 (AC-II) being conserved in *A. thaliana* and six sequences from *Z. mays* (Supplementary Table S1). When the groups of genes were analyzed separately, the GS1b AC box 1 (AC-II) was also found in one of the *B. distachyon* branches. Interestingly, the HB05/HB12 BS2 was also detected in two branches from *H. vulgare*, whereas the PY-box was determined in two branches from *H. vulgare* and one branch from *B. distachyon.*

**Figure 6:**
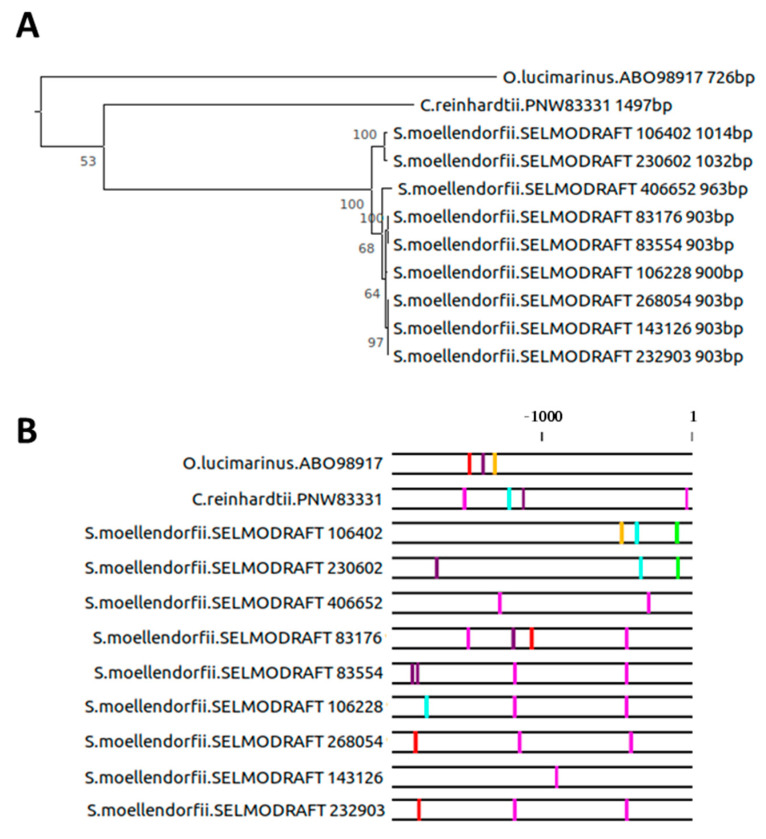
Phylogenic analyses of *SAG12* and its promoter region *Ostreococcus lucimarinus*, *Chlamydomonas reinhardtii*, and *Selaginella moellendorffii*. (**A**) Phylogeny of *SAG12* coding sequence indicating species, gene identification, and sequence length and (**B**) 2000 bp region adjacent to the ATG start codon annotated for the presence of W-boxes (T/C TGAC T/C), A-boxes (TACGTA), T-boxes (AACGTT), C-boxes (GACGTA), and G-boxes (CACGTA). W-boxes in sense (Pink), W-boxes in anti-sense (Purple), A-boxes (Green), T-boxes (Cyan), C-boxes (Yellow), and G-boxes (Red).

The presence of gene duplications and the development of alternative *cis*-regulatory contexts is evident as early as the transition from aquatic unicellular organisms to multicellular land plants (Figure 6A). A single *SAG12* sequence was present in each of the two representatives of the algae, which underwent multiple duplication events during evolution. The first duplication event gave rise to two branches in the tree of *Selaginella moellendorffii SAG12* genes, the smaller one containing two sequences and the larger one containing seven. Their promoter regions (Figure 6B) changed differently during evolution, with characteristic A- and T-boxes close to the start codon present in the smaller branch and a W-box further upstream, close to the −500 bp region in the larger branch. Additional G-, T-, and W-boxes at various positions are consistent with the more dynamic evolution of these promoter regions compared with their relatively stable coding sequences. The high similarity between the gene promoter regions, coding sequences, and lengths indicates that potentially two functionally similar *SAG12* orthologs in different promoter contexts exist in *Selaginella moellendorffii*. This was supported by the Softberry NsiteM-PL analysis (Supplementary Table S1). No conserved motifs were found between *A. thaliana* and *S. moellendorffii* when all sequences were compared together. However, when the two branches were separated, a relatively large number of conserved binding motifs was found within the *S. moellendorffii* family. Eight out of 22 binding motifs from the smaller branch were present in *A. thaliana*, whereas only two out of 15 were present in *A. thaliana* from the larger branch. Interestingly, both the AS1-AS2 BS II (BP Y) and the GARP BS 1 were found in at least one of the two *S. moellendorffii* sequences from the smaller branch.

Additionally, we used the HOMER v4.11 software to search for known and potential unknown motifs over-represented in the collection of 2000 bp promoter regions of this study. The collection of all 2000 bp promoter regions from Arabidopsis TAIR10 release was used as a background. Nineteen known motifs with lengths from eight to 15 nucleotides were recognized, 11 of them present in the *AtSAG12* promoter (Supplemental Table S5). HOMER orders all sequences by “rank” according to the *p*-value for each conserved motif. Known motifs with rank 1, 4, 7, 8, and 17 showed partial or complete overlap, also overlapping with three of the five A-boxes in the *AtSAG12* promoter. Motif 8 was also found in 94.44% of the promoter regions used in this study. This observation supports the notion of possible accessory function of these motifs, conserved within the plant kingdom. Still, no known motifs were located within the main regulatory region recognized by Noh and Amasino [14]. We screened further for unknown motifs with lengths of 6, 8, 10, 12, 14, and 16 nucleotides and discovered between 12 and 24 over-represented motifs. A total of 13 motifs from all groups were found within the *AtSAG12* promoter. The six-nucleotide long motif with rank 3 (present in 83.33% of the sequences) was located within the main regulatory region recognized by Noh and Amasino [14], together with the eight-nucleotide long motif with rank 2 (present in 43.33% of the sequences) and the 12-nucleotide long motif with rank 4 (present in 17.78% of the sequences). These represent good candidate sequences for further analysis for functional *cis*-acting motifs.

### 2.4. Intron–Exon Structure of the AtSAG12 Orthologs

In addition, we examined the intron–exon structure of the orthologs of *AtSAG12* from the data available in the Ensembl Plants website (Supplementary Table S2). Most genes comprised two exons and one intron, with more than two-fold enrichment compared to the next most prevalent group of three exons and two introns, to which *AtSAG12* belongs (Supplementary Figure S2A). Furthermore, most genes have two or more exons, with only a few examples with one exon present in the dataset. The one-exon orthologs were mostly genes with length of ~500bp, most likely comprising partial sequences. The majority of genes have a conserved sequence length of 1–1.25 kb corresponding to a transcript length of ~ 1 kb and translation length of 300–350 amino acids (Supplementary Figure S2B–D). Notably, while the distribution of gene lengths is slightly skewed in favor of sequences larger than the median of 1.15 kb and exon number larger than the median of two exons, transcript length shows approximate normal distribution around the median of 1027 bp. Translation products are slightly skewed in favor of sequences with less than the median of 340 amino acids (Supplementary Figure S2B–D). An outlier in this dataset is the *C. reinhardtii* PNW83331 gene with 13 exons, a gene length of 7.87 kb, a transcript length of 2248 bp, and a translation product of 498 amino acids. These data indicate that gaining gene length via intron expansion and incorporation of additional introns is more prevalent. While transcript length appears conserved, loss of amino acids is slightly more predominant than gain in our dataset. 

## 3. Discussion

The aim of this work was to examine coding sequences and promoter regions of a known senescence-associated gene, *AtSAG12*. This gene was chosen because of its relatively well-understood promoter region, which is targeted by WRKY transcription factors in *Arabidopsis*, and its wide usage as a marker gene to quantify senescence throughout the plant kingdom. We have identified *cis*-regulatory sequences (A-boxes) that are located close but outside of an important regulatory region of its promoter and that probably occurred not by chance and, thus, are under positive selection (Figure 1B). A-boxes are typically targeted by bZIP transcription factors that are involved in the transmission of upstream signals into gene expression changes that are often mediated by phosphorylation, dimerization, and/or translocation into the nucleus. Transcriptional changes might be involved in the responsiveness of *AtSAG12* to cytokinin, auxin, and other signals in order to integrate them with age- and stress-related gene expression. An interplay between WRKY and bZIP but also unknown transcription factors might, thus, be responsible for the intricate regulatory network that fine tunes the expression of the *SAG12* gene during *A. thaliana* leaf senescence. Additionally, we have detected transcription factor binding sites for the AS1-AS2 nuclear protein complex involved in leaf morphogenesis [34] and GARP transcription factors involved in nutrient signaling and plant development [35]; these sites also lie directly next to, but outside of, the main regulatory region of *AtSAG12* and show some level of conservation throughout the plant kingdom. Our findings suggest that a relatively large number of potential *cis*-regulatory motifs are involved in the fine tuning of *AtSAG12* gene expression (Supplementary Table S1), in concordance with the notion that, on average, any Arabidopsis gene is targeted by 25–75 binding events of transcription factors [7,20]. Which of these sites/factors are functionally active and contribute to the expression pattern remains to be elucidated. However, the senescence-associated expression appears to be conserved over the plant kingdom, as expression studies in many higher plants reveal that at least one of the gene copies of the SAG12 family is upregulated in senescent tissue (Supplemental Table S4). Consistent with these findings, the expression of the P*_AtSAG12_:IPT* construct in transgenic plants of a wide range of species is similar and highly conserved (Supplemental Table S3). The autoregulatory feedback regulation between senescence-associated *SAG12* promoter-driven cytokinin production and subsequent inhibition of the *SAG12* promoter-driven expression (Figure 1C) also appears to work with the *trans* factors of the transformed plants indicating a basal conservation of the overall regulatory cues even though the conservation of the *cis* elements appears to be not obvious when screening for perfect matches of 6 letter words.

Substantial differences in *cis*-regulatory elements in the *SAG12* promoter have previously been identified in related Arabidopsis species. *SAG12* expression has been noted in *A. lyrata* roots, but multiple datasets suggest that this is not the case in *A. thaliana*. However, James et al. [30] have recently described the possible activity of *SAG12* in *A. thaliana* roots and discuss its expression in the root stele during reproduction under low nitrogen conditions. Developmental differences in gene expression in roots might, thus, be assigned to differences in experimental sampling conditions. Moreover, different gene expression patterns might be indicators for the different regulation of gene expression attributable to changes in the *cis*-elements of their promoter regions or in *trans*, meaning that the amount of transcription factor, its tissue-specific expression, or its target specificity of binding to the promoter regions might have changed. A lower *SAG12* expression level in *A. lyrata* is consistent with the loss of the enhancer region described by Noh and Amasino [14]. The distance and the orientation of a promoter element relative to the transcription start site influences gene activity [36]; the much lower expression level in *A. lyrata* flowers [28] compared with those of *A. thaliana* [29] is in accordance with this observation. Unfortunately, no expression profile for senescent *A. halleri* is available. Honjo and Kudoh [37] have reviewed some of the main ecological differences between the three Arabidopsis lineages and point out that *A. thaliana* is mostly an inbreeding annual monocarpic plant, whereas *A. lyrata* and *A. halleri* are in general outbreeding perennials. The signaling involved in senescence is, therefore, expected to be under different evolutionary pressure; *A. thaliana* as an annual plant undergoes monocarpic senescence after anthesis, whereas *A. lyrata* and *A. halleri* as perennial plants undergo sequential and stress-induced senescence. Furthermore, *A. thaliana* seems to be native to warmer climates closer to the tropics compared with the other two species. This might be reflected in the observed differences in the three promoter regions. Indeed, genome-wide analyses provide considerable support that expression differences for individual genes have often evolved in *cis*-elements [5].

Here, we have used transcription factor binding sites (W-boxes, etc.) that are structurally conserved across the plant kingdom, although no direct experimental evidence exists that these are really targeted by certain transcription factors in individual species. Transcription factors may bind to relaxed motifs hidden in the promoters, and from chromatin immunoprecipitation studies using for example bZIP factors, we know that only shorter core motifs are essential, whereas neighboring nucleotides can vary [38,39]. Interestingly, whole-genome-targeted chromatin immunoprecipitation studies have also revealed only a limited conformity between experimentally established genomic DNA-binding locations and associated transcriptional changes. The overlap in early studies involving *A. thaliana* ranges from 2 to 25 % [20]. Apparently, many binding events have no or little impact on gene expression [20], questioning the explicit importance of the putative binding sites discussed here. Furthermore, cysteine proteases are large gene families in most plants, and senescence-associated gene functions might have been taken over by distant family members; this, however, will not affect our conclusions concerning promoter diversity in highly conserved genes. Further elucidation of the influence of the A-box elements on gene expression during senescence in *A. thaliana* and detailed expression profiles of leaf senescence in other species included in this study should throw light on the influence of *cis*-regulatory elements on gene expression and the interplay between *cis* elements and *trans* factors in adaptive evolution with regard to *SAG12* and its orthologs. As our study is purely bioinformatical, it should serve as a background for future research in this area.

However, an alarming finding of our research was the inability of the Softberry algorithms to recognize the perfectly matched A-boxes present in the *AtSAG12* −2000 bp region. However, out of the more than 200 entries for experimentally proven A-, C-, or G-boxes in various plant species used in the database, none of these experimentally proven A-box motifs appears to match the A-box motif in the promoter of the *AtSAG12* to 100%. Most likely not only the consensus but also the flanking regions appear to be included in the search algorithm. Furthermore, it is possible that due to mismatches in the regions containing the A-boxes when comparing to entries in the database, the algorithm assumed random occurrence. By showing that random occurrence is highly unlikely, we demonstrated that even a finely tuned algorithm such as Softberry Nsite can benefit from further adjustments. The accuracy of such algorithms in predicting regulatory regions on whole genome sequences was also deemed insufficient by the authors [26]. Further research verifying the activity of these regulatory elements can solidify this statement. 

A strength of the HOMER v4.11 software is the usage of position weight matrices when looking for known motifs. This allows certain mismatches to be included in the recognition process. On the other hand, the recognition process is largely dependent on the background sequences against which the comparison is made. Finding an adequate set of sequences is not always possible. Even though the package allows for the generation of a random sequence by “scrambling” the original set, the nucleotide sequences within a genome are not random, and such an approach will certainly lead to a bias. In the present study HOMER v4.11 was used to recognize both known and unknown motifs, strengthening the notion that the A-boxes found within the *AtSAG12* promoter may have some additional regulatory function, maybe in stress cross talk, as these *cis*-elements appear to be at least partially conserved within other species. Interestingly, only three of the five motifs were matched in the context of larger known motifs, pointing at the inability of the software to pick perfectly matching motifs in any case. Furthermore, three over-represented unknown motifs were proposed by the software, which are located within the regulatory region recognized by Noh and Amasino [14]. Thus, the approach used by HOMER v4.11 proved to be more adequate for the purpose of recognizing both known and unknown motifs that are over-represented within the promoter regions of *AtSAG12* orthologs.

Four *SAG12* copies with distinct promoter regions were identified in *B. napus* (Figure 3). Previously, Noh and Amasino [40] had discovered two functional orthologs of *AtSAG12* in *B. napus* and named them *BnSAG12-1* and *BnSAG12-2*. *BnSAG12-1* is related closely to the *BnaA02g24130D*–*BnaC02g31910D* pair, whereas *BnSAG12-2* corresponds to the *BnaA06g40240D*–*BnaC07g18750D* pair. Incubation of the functional conserved motif of approx. 310 bp from *B. napus* with protein extract from senescing *A. thaliana* leaves led to binding activity, but this was much weaker than that of the *A. thaliana* region. This genomic region includes the main regulatory elements described by Noh and Amasino [14]; the whole 3.11 kbp promoter region coupled to the GUS reporter and transformed into *A. thaliana* revealed senescence-specific expression. This inter-species activity was, however, much weaker than expected from expression levels in *B. napus*. Indeed, only a single complete A-box was left in *BnaA06g40240D*. This means that the other A-boxes are essentially lost in other Brassica species. bZIP transcription factors, however, possibly also bind to non-complete A-boxes, and mutations of these A-boxes might be the reason for the weaker binding by *A. thaliana* transcription factors. Furthermore, discrepancies in inter-species activity suggest that the conserved AS1-AS2 BS II (BP Y) motif, recognized by Softberry NsiteM-PL, is not the main regulatory element responsible for regulating senescence-specific gene expression. Even though the senescence-specific transcriptional profile of *B. napus SAG12s* closely resembles their ortholog in *A. thaliana*, the molecular *cis*-acting regulatory regions appear distinct, whereas the gene coding sequence maintains high similarity. The two *B. napus* families, thus, share similar expression patterns of *SAG12* but are under the control of different regulatory elements [40]. Potential stabilizing selection on gene expression via *cis-trans* compensation is a suitable explanation.

The larger family of *SAG12* genes in poplar apparently evolved only recently (Figure 4), illustrating that gene duplication events may not only be important for the evolution of the coding sequence, but also allow the development of similar genes in novel regulatory contexts. Together, the strong divergence of promoter *cis*-regulatory elements between the three grass species (Figure 5) precludes conclusions on conserved promoter elements. We speculate that duplication events both including and excluding the 2000 bp promoter region took place. Thus, the much weaker evolutionary pressure on promoter elements (e.g., compared with coding sequences) together with the appearance of new gene copies in new regulatory contexts brought rise to a large variety of putative genes and *cis*-regulatory contexts, further enriching the evolutionary potential of these plant species. The transition from water to land also coincides with a drastic increase in orthologs of *AtSAG12* in *S. moellendorffii,* compared with *C. reinhardtii* and *O. lucimarinus* (Figure 6). The first *SAG12* gene in unicellular algae might have evolved as part of stress responses, since a strikingly senescence-like response on the removal of light and nitrogen source is seen in cultures of *Chlorella protothecoides*. Cells turn yellow, accompanied by changes in protein and enzyme activities that resemble those of senescing mesophyll cells [41,42]. Gene duplication events in the plant kingdom have previously been described as a major evolutionary force [43,44,45]. The number of duplicated gene pairs derived from whole genome duplications is known to decline with increased time after the event, whereas the number of local duplications shows no decrease with time, in concordance with our findings. Thus, tandem and proximal duplications might provide a continuous supply of genes useful for plant adaptation. In the *Viridiplantae* clade, 63–92% of gene pairs from whole genome duplications and local duplications have diverged in their gene expression patterns, showing that the differential gene expression of pairs is potentially a major source of variation in plants [46]. This analysis poses the questions as to whether most local gene duplication events happen at random or around particular “hotspots” at which evolutionarily “active” genomic regions tend to produce variation, and whether different environmental stress factors influence the appearance of local and global gene duplication events. 

We found that the evolution of the coding sequences of the *AtSAG12* orthologs is driven primarily by intron expansion and insertion. Intron–exon evolution in eukaryotes is a well-studied and widely discussed topic with a comprehensive review provided by Rogozin et al. [47]. They recognize a correlation between intron density and size, stating that with intron density of up to three introns per kilobase, introns tend to be relatively short [47]. Concordantly, we find that the majority of sequences in our study comprise two exons and one short intron or three exons and short introns. Furthermore, a high rate of intron gain was described for paralogous gene pairs in Arabidopsis [48], while a low rate of intron gain was observed in plastid derived genes in plants [49]. Therefore, our findings do not diverge from the expected patterns of evolution of plant orthologs. In contrast, the conservation of the exon–intron structure and gene length also supports conservation of function and possibly even intron-based regulation, which we did not analyze in more detail.

The *cis*-regulatory landscape involved in the expression of *AtSAG12* and its orthologs is further influenced by the diversification of *trans* acting factors, such as the WRKY and bZIP transcription factor families. Perhaps the most drastic example for plants adapting to novel environments is the early colonization of land by plants. Environmental pressure for the development of novel mechanisms to cope with a myriad of stress factors not present in pre-existing aquatic ecosystems arguably peaked at this period. This brought in large diversifications in two of the main transcription factor families involved in the plant stress response: WRKY and bZIP [50,51,52,53,54,55]. From a typical occurrence of 1–3 WRKY genes in green algae, such as *C. reinhardtii* and *O. lucimarinus*, the spike moss *S. moellendorffii* has developed most groups of WRKY transcription factors found in modern-day flowering plants [54]. The situation with the bZIPs is more complicated. Whereas WRKYs are mostly plant-specific, bZIP transcription factors are found in all eukaryotes. The transition of plants from water to land coincides with major developments in a number of pre-existing bZIP groups involved in the light response, nitrogen/carbon balance control, ion responses, and biotic stress responses [52]. The increase in the number of genes from each of the two families during land colonization is further amplified by the potential of both family members to form homo- and heterodimers, further diversifying the regulatory interactions that evolved during this period. A substantial overlap also occurred in the functions of the two families, specifically in pathogen response, the hormonal activity of JA, SA, and ABA, and leaf senescence [3,25,53]. This strengthens the idea that the co-evolution of these two families have, at least partially, shaped stress response signaling and *SAG12* expression in the plant kingdom. 

A research on budding yeast uncovered that even though substitutions of nucleotides comprising transcription factor binding sites is slower than the surrounding regions, it is not zero [56]. Similar conservation of amino acids involved in the formation of the protein–DNA complex of a transcription factor corresponded to a correlation between the evolution of transcription factors and their binding sites [56]. Shultzaberger and coworkers [57] demonstrated that a large range of binding preferences, information contents, and activities can be achieved with a few mutations of a transcription factor sequence, suggesting that transcriptional regulatory networks are easily adaptable. Therefore, co-evolution of transcription factors and their binding sites over a long period of time can lead to distinct differences in *cis*-regulatory regions of a gene, without significant changes in transcriptional activity. Furthermore, Ching et al. [58] recognized that some binding sites may retain function, despite mutations in degenerate positions with little impact on transcription factor binding. While different transcription factors may bind sequences with relaxed specificity at certain positions, usage of exact words may not be the best strategy for recognizing conserved *cis*-regulatory elements. A study in *E. coli* brought up the idea that a decrease in the free energy of association due to a mutation in one site could be compensated for by an increase in the binding energy at another [59]. It is, therefore, expected that the turnover of transcription factor binding sites is faster than the evolution of the corresponding transcription factors. Lusk and Eisen [60] estimated that the expected half-life for a binding site of a typical *D. melanogaster* transcription factor is around 50 to 100 million years. It is not surprising that when the genome-wide binding of six transcription factors were compared between *D. melanogaster* and *D. yakuba*, the factor specific variation in binding was driven to a large extend by the gain and loss of recognition sequences for a given factor [61]. An example of the importance of such variation in gene expression for evolutionary adaptation was demonstrated by Fay et al. [62] when comparing gene expression in nine natural isolates of *S. cerevisae* under stressful conditions. All these lines of evidence suggest that the lack of conservation among transcription factor binding sites identity, position, and frequency in such evolutionary timescales as in the present study is an expected phenomenon. A glaring weakness in the present study is the usage of strict six letter words when searching for conserved *cis*-regulatory elements. A rapid turnover of transcription factor binding sites is expected to bring notable differences in even closely related species, while mutations in the corresponding transcription factors can make enhancer sequences of distant relatives virtually incomparable, while retaining the original functionality of the gene. Furthermore, differences in gene expression profile, driven by *cis*-regulatory element turnover and transcription factor evolution, can potentially contribute to individual fitness under stressful environments.

Although our analysis was focused only on potential *cis*-regulatory changes in the gene promoters, it is clear that changes also take place in *trans* (such as the different expression or binding specificity of a transcription factor). This is based on the observation that, despite the senescence-associated expression of *SAG12* in other species, e.g., *BnaA06g40240D* [40] and Solyc02g076910 [63,64], some regulatory factors in *trans*, such as WRKY or bZIP transcription factors, lost their target sites. As a consequence, other novel transcription factors are probably involved in *SAG12* expression regulation. As in *Brassica napus*, the two pairs of *SAG12* genes and promoters are more similar within the two pairs than between the two pairs, each pair appears to share an isolated evolutionary history and possible exposure to different sets of trans regulators. Nevertheless, causal *cis*-regulatory changes are more commonly found in “structural” genes such as *SAG12* that are involved in catalyzing enzymatic reactions rather than regulatory genes, which is in concordance with our own observations [5]. In robust gene networks, *trans* factors have often been found to make large intraspecific contributions, as is consistent with the loss or occurrence of W- or A-boxes between species [5].

Taken together, our work highlights the adaptive potential of *cis*-regulatory transcription factor target sites compared with gene body evolution for a single senescence-associated gene. Within the context of gene network regulation, “peripheral” network genes such as *SAG12* as a structural, rather than as a regulatory gene, have been reported to be highly variable in their *cis*-regulatory features [4]. Further research into the interactions between *cis*-elements, binding factors, and gene regulation and activity is crucial for a deeper understanding of the processes that allow plants to adapt to changing environmental conditions in the long term. Since our current breeding methods demand a large genetic variation in our seed stocks, an understanding of the mechanisms that plants use to generate variation on a local and global scale is crucial for our efforts to breed crops suitable for a continuous changing climate.

## 4. Materials and Methods

### 4.1. Sequence Analyses

The complete sequence of *A. thaliana* was downloaded, together with gene annotations, from the TAIR10 release. A custom python script was used to find the locations of all W-boxes (T/CTGACT/C), A-boxes (TACGTA), T-boxes (AACGTT), C-boxes (GACGTA), and G-boxes (CACGTA). We used custom python scripts to extract the 2000 bp regions immediately upstream of the ATG start codons of all genes. We annotated these regions and counted the number of W-boxes, A-boxes, T-boxes, C-boxes, and G-boxes present. The graphical representation for the distribution of transcription factor binding sites was performed with ggplot2 in R. 

To calculate the chance for random occurrence of a transcription factor binding site the genome was separated into 2000 bp long non-overlapping fragments. The GC content of each fragment was calculated and saved using a custom python script. Dinucleotide frequencies were calculated separately for the whole genome and for regions with GC content of up to 2% deviation from that of the AtSAG12 promoter. The chance for random appearance of a transcription factor binding motif was treated as a first order Markov chain, where the appearance of every nucleotide is influenced by the previous one. The appearance of the first nucleotide of the sequence was treated as an independent event. Therefore, the chance for occurrence of an, e.g., A-box, was calculated as follows:

pAbox=pT×pTA×pAC×pCG×pGT×pTA

### 4.2. Phylogentic Trees

For the phylogenetic tree analysis, we searched the Ensembl Plants database release 45 for orthologs of *AtSAG12* (AT5G45890) and selected the following sequences for further analyses: *Ostreococcus lucimarinus* (1 sequence), *Chlamydomonas reinhardtii* (1 sequence), *Selaginella moellendorffii* (9 sequences), *Brachypodium distachyon* (8 sequences), *Hordeum vulgare* (15 sequences), *Zea mays* (12 sequences), *Dioscorea rotundata* (9 sequences), *Amborella trichopoda* (5 sequences), *Vitis vinifera* (7 sequences), *Prunus persica* (4 sequences), *Arabidopsis lyrata* (1 sequence), *Arabidopsis halleri* (1 sequence), *Arabidopsis thaliana* (1 sequence), *Brassica napus* (4 sequences), *Beta vulgaris* (1 sequence), *Solanum tuberosum* (1 sequence), *Solanum lycopersicum* (1 sequence), *Actinidia chinensis* (1 sequence), *Theobroma cacao* (1 sequence), *Gossypium raimondii* (1 sequence), *Manihot esculenta* (1 sequence), and *Populus trichocarpa* (7 sequences). Our criterion was that sequence similarity must not be below 30%. Because of the large number of sequences meeting this criterion, we chose a small subset of representatives that covered lower to higher plants, perennials and annuals, and temperate to tropical environments. Thus, we obtained sequences from 22 species in total. We downloaded the coding sequences plus the adjacent regions containing 2000 bp upstream of the ATG codon and recorded the available data on exon number, gene length, transcript length, translation length, and number of splice variants. When more than one splice variant was available, only the top candidate recognized in the database was recorded. We separated the protein coding sequence for alignment and phylogenetic tree analysis and the 2000 bp region adjacent to the ATG start codon for a comparison of regulatory elements. The coding sequences were aligned using MEGAX Version 10.0.5 with ClustalW on default settings. The phylogenetic tree was then constructed using the maximum likelihood statistical method with the general time reversible substitution model, gamma-distributed mutation rates, and the nearest neighbor interchange (NNI) heuristic method, at default settings. Statistical evaluation was performed via bootstrapping with 100 repetitions. Promoter region annotation was carried out using custom motifs on CLC Main Workbench 8.1. Visualization was achieved via the GNU Image Manipulation Program. 

### 4.3. cis-Element Analyses

To find alternative transcription factor binding sites, we used the online tools provided by Softberry, namely, Nsite-PL for conserved binding sites in *A. thaliana* and NsiteM-PL for conserved binding sites, when comparing promoter sequences from different species. The minimum similarity level in percent was set to 100. When no conserved sequences were found between *A. thaliana* and the whole group of gene promoters from a single species, the group was divided into subgroups based on the similarity of their coding sequences.

Softberry Nsite uses a probabilistic model to assess the statistical significance of motif similarity. Assuming binomial distribution for matches and mismatches, the algorithm calculates the probability for random occurrence of a segment with defined length and number of mismatches in variable positions [26]. The database that is used for comparison is the RegSite Database of Plant Regulatory Elements (http://www.softberry.com/berry.phtml?topic=regsitelist (accessed on 1 February 2021)). In this database, there are 99 entries for experimentally proven W-boxes and more than 200 entries for experimentally proven A-, C-, or G-boxes in various plant species; however, none of these experimentally proven A-box sequences appears to match the A-box motif of *AtSAG12* to 100%. 

HOMER v4.11 [65] was used to recognize potential regulatory motifs conserved within our collection of promoter sequences. The list of promoter sequences of all *AtSAG12* orthologs was put in FASTA format and compared to the list of all 2000 bp promoter sequences from Arabidopsis, downloaded from the TAIR10 release. The presence of unknown motifs with length of 6, 8, 10, 12, 14, and 16 nucleotides was recorded along with all known motifs of various lengths. HOMER utilizes the JASPAR database for finding known motifs. *findMotifs.pl* was used with default settings to locate both known and unknown motifs. The lengths for unknown motifs was set with the *-len* setting. After the position weight matrices were obtained for all motifs, their presence within the *AtSAG12* promoter was established with the *-find* setting of *findMotifs.pl.*

### 4.4. Recourses Used for Gene Expression Analyses

Expression data were mostly obtained from the Expression Atlas (http://ebi.ac.uk (accessed on 1 November 2020)), which contains 972 plant experiments across different species and under different biological conditions. Moreover, Breeze and colleagues did a high-resolution temporal profiling of transcripts during Arabidopsis leaf senescence [3], which we used in addition for expression analyses in *Arabidopsis thaliana.* Data are available at the NCBI Gene Expression Omnibus (https://www.ncbi.nlm.nih.gov/geo/query/acc.cgi?acc=GSE22982 (accessed on 1 November 2020)). 

## Data Availability

Not applicable.

## Supplementary Materials

The following are available online at https://www.mdpi.com/article/10.3390/plants10071380/s1, **Figure S1**: Phylogenetic relationship between all SAG12 genes analyzed in this study. **Figure S2**: Histograms of (A) exon number; (B) gene length; (C) transcript length; (D) translation length of all *SAG12* genes analyzed in this study. **Figure S3**: Sequences alignment of the promoter regions of the Arabidopsis species. **Table S1**: Nsite-PL analysis of conserved binding sites in 2000 bp region upstream from the start codon of SAG12 in *A. thaliana*. From left to right the table contains: species in which the TFBS was first identified (Species); name of the TFBS (TFBS); transcription factor that interacts with the binding site (BF); the strand in which the TFBS resides (strand); the start (Start) and end (End) position of the TFBS; the conserved sequence of the TFBS (Sequence). NsiteM-PL analysis of TFBS conserved between multiple sequences. On the left side, from left to right the tables contain: motif identification number (Motif); species in which the TFBS was first identified (Species); name of the TFBS (TFBS); transcription factor that interacts with the binding site (BF); conserved sequence of the TFBS (Sequence). On the right side, the presence of a motif within a sequence is identified with “+”, while its absence with “.”. **Table S2**: Exon number, gene length, transcript length, translation length, and number of splice variants of all SAG12 genes analyzed in this study, as present in the Ensembl Plants database. **Table S3**: Expression of P*_AtSAG12_*:*IPT* in other plant species. **Table S4**: Senescence-associated expression of *SAG12* in other plant species. **Table S5**: HOMER v4.11 analysis for over-represented regulatory motifs within the promoter regions of all *AtSAG12* orthologs.
