# Supplementary material for: Disparate Dynamics of Gene Body and cis-Regulatory Element Evolution Illustrated for the Senescence-Associated Cysteine Protease Gene SAG12 of Plants
Source: Plants (Basel). 2021 Jul 6;10(7):1380. doi: 10.3390/plants10071380 (PMC8309469; doi:10.3390/plants10071380)
Supplement: Supplementary file 1 [file plants-10-01380-s001.zip › Supplementary Figure S3.pdf]

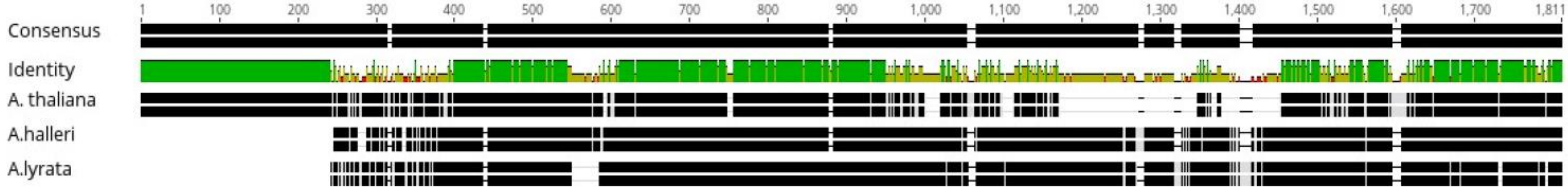

Figure S3. Sequence alignments of the 1500bp promoter regions from the Arabidopsis lineage. Here, position 1 corresponds to the position -1811 bp upstream of the ATG start codon of the *A. thaliana SAG12* gene. This alignment was done using Geneiousversion 2021.1 with ClustalOmega 1.2.2. Sequences were grouped by similarity using fast clustering (mBedalgorithm) with cluster size for mBedguide trees set at 100.
