# Supplementary figures and images for: Disparate Dynamics of Gene Body and cis-Regulatory Element Evolution Illustrated for the Senescence-Associated Cysteine Protease Gene SAG12 of Plants
Source: Plants (Basel). 2021 Jul 6;10(7):1380. doi: 10.3390/plants10071380 (PMC8309469; doi:10.3390/plants10071380)

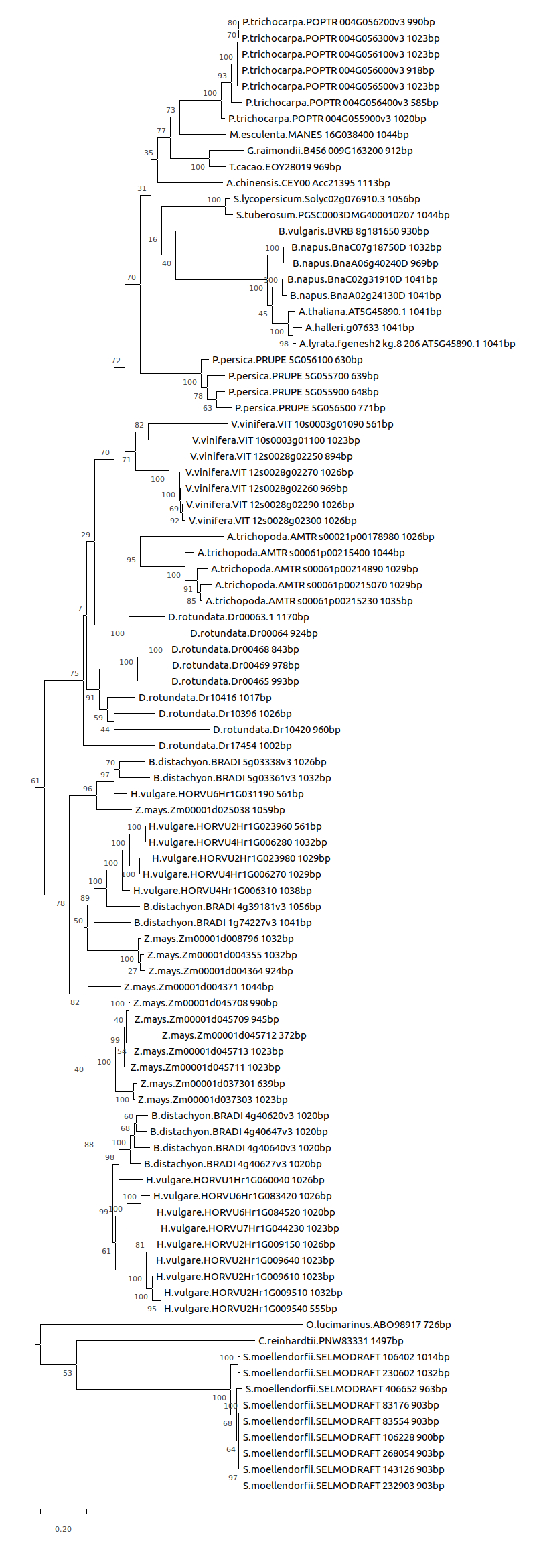

Supplement: Supplementary file 1 [file plants-10-01380-s001.zip › Supplementary Figure S1.jpg]

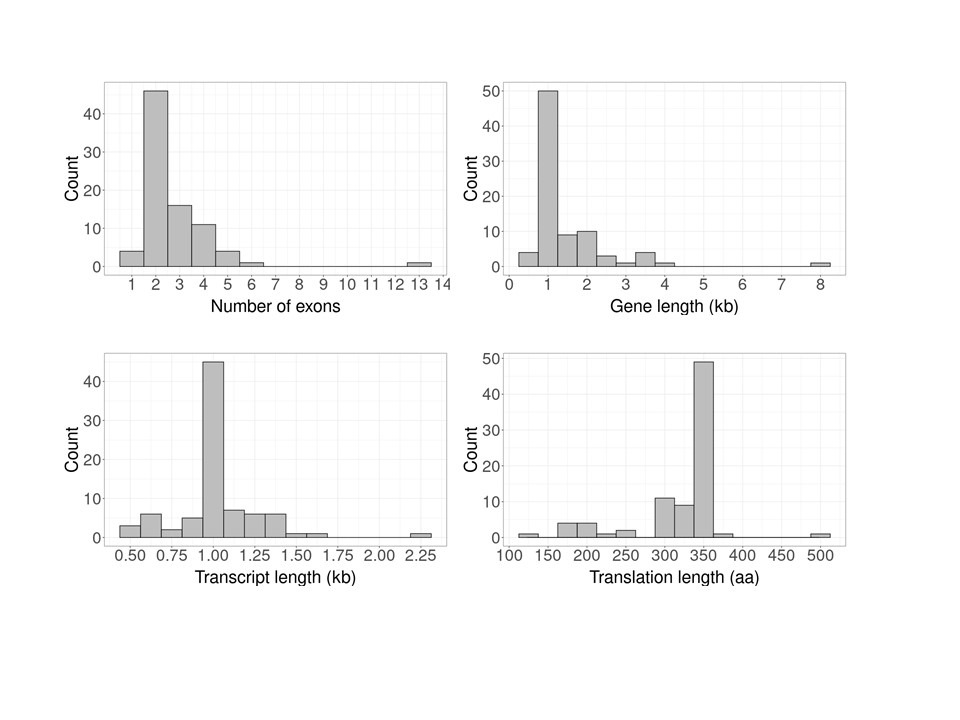

Supplement: Supplementary file 1 [file plants-10-01380-s001.zip › Supplementary Figure S2.jpg]
